# Supplementary material for: Learning curve and surgical outcome of robotic assisted colorectal surgery with ERAS program
Source: Sci Rep. 2022 Nov 29;12:20566. doi: 10.1038/s41598-022-24665-w (PMC9709162; doi:10.1038/s41598-022-24665-w)
Supplement: Supplementary file 2 — Supplementary Information 2. [file 41598_2022_24665_MOESM2_ESM.zip › RAL Raw data-Ñ[▒K/118/OP N_ñw╝╨░O▒Kñσ.pdf]

W52 / W52

臺中榮民總醫院
手術室護理記錄

[redacted]

基本資料

- 室別： 開刀房-17室，手術科別： CRS，手術類別： 常規，日期： 2021/10/13
- 進入時間： 0800 ； 麻醉開始時間： 0815 ； Time Out時間： 0940 ； 劃刀時間： 0941 ； 手術結束時間： 1550 送出時間： 1610 ；
- 麻醉方式： GE

  診斷： Malignant tumor of rectum

  手術名稱： Robot Assisted Low Anterior Resection

  手術局部用藥： nil

  術前預防性抗生素：
    - 抗生素名稱一： Veterin 1GM IVD(由麻醉科給予)
    - 時間： 0935
    - 上次簽章: 劉雅汶

  ，追加預防性抗生素：
    - 抗生素名稱一： Veterin 1GM IVD(由麻醉科給予)
    - 時間： 1335
    - 上次簽章: 黃彥翔
- 手術醫師： 蔣鋒帆，陳璿夫，陳凱祥
- 麻醉醫師： VS 陳永銘
-
    - 刷手護士A ： 黃彥翔，時間： 0800，刷手護士B ： 鄭鈞躍，時間： 0800，接替刷手一： 張秀敏，時間： 1200，接替刷手二： 張秀敏/許芷瑄，時間： 1500
    - 流動護士A ： 劉雅汶，時間： 0800，流動護士B ： 詹佳穎，時間： 0800，接替流動一： 黃彥翔，時間： 1230，接替流動二： 陳盈妤，時間： 1500

皮膚狀況

A-V Shunt： 無

術前： 不完整

- 7. 破皮

部位(術前)：

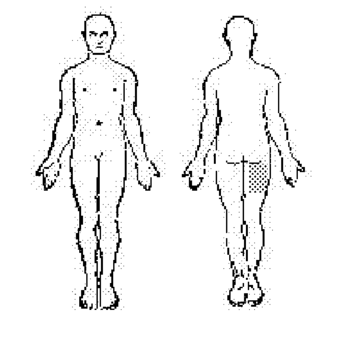

右大腿約5x5cm

記錄者： 上次簽章: 陳盈妤

術後： 不完整

- 7. 破皮

部位(術後)：

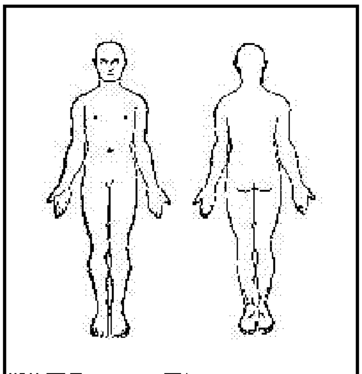

術後薦骨3cm×3cm壓紅。

記錄者： 上次簽章: 陳盈妤

手術準備

手術臥式 單一手術臥式

-
  - 手術臥式： 截石臥式
  - 使用附件： 溫毯 38 ℃，矽膠枕頭，腳架
  - 皮膚準備： 10%Aq Betadine，Hibiscrub，2% Chlorhexidine
  - 電燒： 使用電燒: 電極片位置： 其它： 電燒迴路軟墊，記錄者： 上次簽章: 劉雅汶

止血帶T： 無

植入物

- 植入物名稱/號碼/數量/部位： Ethicon Curved Intraluminal Stapler EEA 29mm/*1/colon
  Endowrist stapler 45mm green/*2/colon

, 記錄者： 上次簽章: 黃彥翔

標本送檢

- 細菌培養 無
- 細胞學 無
- 冰凍切片 無
- 病理標本
  組織名稱一： distal cut end
  ,
  組織名稱二： IMA root
  ,
  組織名稱三： rectum
  , 更多組織名稱
  - 組織名稱四： omentum
- 截肢 無

標本交付 標本： 2 袋，標本： 0 盒，標本： 2 瓶，細胞學： 0 管，Culture Tube： 0 管，被交付醫師： CR 陳瑢夫，

- 記錄者： 上次簽章: 陳盈妤
- 時間： 1550

傷口術後敷料

傷口敷料： 傷口敷料： 無

- 請於圖上標示部位：

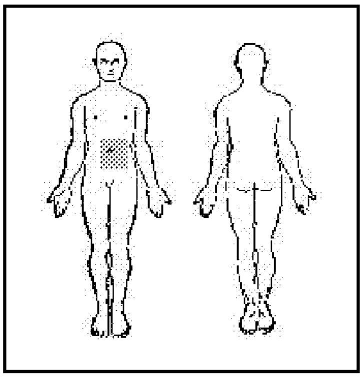

- 傷口敷料： trocar傷口:軟性敷料

導管

導管	天數	導管資訊	插入日期	插入時間	更換日期	拔除日期	拔除時間	拔除原因	預計拔除/更換	預計拔除/更換日期
Epidural PCA(1)	1	部位:硬脊膜下;	2021/10/13	0835						
Penrose Drain(1)	1	號碼:12 mm.; 部位:骨盆腔;	2021/10/13	1400						
Double Lumen CVC(1)	1	號碼:7 FR; 部位:; 象限:RU; 註記:fix15 cm;	2021/10/13	0830						
Foley(1)	1	號碼:16 Fr.; 部位:膀胱; 註記:balloon 10c.c;	2021/10/13	0850						

用物計數

- 手術中出血量： 150 c.c.
- 手術中尿量： 400 c.c.
-
    - 其它： 無

用物計數： 多切口同時縫合

- 1.器械： 正確，2.縫針： 正確，3.刀片： 正確，4.紗布： 正確，5.腹部墊： 未使用，6.紗球： 未使用，7.PEANUTS ： 未使用，8.紗條： 未使用，9.絲帶： 未使用，10.針頭： 未使用，11.Vessel loop： 未使用，12.Boots： 未使用，13.Jelco： 未使用，14.棉條： 未使用，
    - 第一次：
        - 刷手護士 ： 張秀敏
        - 流動護士： 上次簽章: 黃彥翔
        - 時間： 1430

    ,
    - 第二次：
        - 刷手護士 ： 張秀敏
        - 流動護士： 上次簽章: 黃彥翔
        - 時間： 1435

    ,
    - 第三次：
        - 刷手護士 ： 張秀敏
        - 流動護士： 上次簽章: 黃彥翔
        - 時間： 1440

備註

備註： NO.2 davinci 30度 scope

- 記錄者： 上次簽章: 黃彥翔
- 時間： 0800

填寫護理人員 陳盈妤

正式摘要

W52 / W52

doctorRank:B
